# Structural and functional insight into serine hydroxymethyltransferase from *Helicobacter pylori*

**DOI:** 10.1371/journal.pone.0208850

**Published:** 2018-12-14

**Authors:** Andreea Sodolescu, Cyril Dian, Laurent Terradot, Latifa Bouzhir-Sima, Roxane Lestini, Hannu Myllykallio, Stéphane Skouloubris, Ursula Liebl

**Affiliations:** 1 Laboratory of Optics and Biosciences, Ecole polytechnique, CNRS, INSERM, Université Paris Saclay, Palaiseau, France; 2 Institute for Integrative Biology of the Cell, CEA, CNRS, Université Paris Saclay, Gif-sur-Yvette, France; 3 UMR 5086 Molecular Microbiology and Structural Biochemistry, Institut de Biologie et Chimie des Protéines, CNRS, Université de Lyon, Lyon, France; 4 Department of Biology, Université Paris-Sud, Université Paris Saclay, Orsay, France; Oswaldo Cruz Foundation, BRAZIL

## Abstract

Serine hydroxymethyltransferase (SHMT), encoded by the *glyA* gene, is a ubiquitous pyridoxal 5’-phosphate (PLP)-dependent enzyme that catalyzes the formation of glycine from serine. The thereby generated 5,10-methylene tetrahydrofolate (MTHF) is a major source of cellular one-carbon units and a key intermediate in thymidylate biosynthesis. While in virtually all eukaryotic and many bacterial systems thymidylate synthase ThyA, SHMT and dihydrofolate reductase (DHFR) are part of the thymidylate/folate cycle, the situation is different in organisms using flavin-dependent thymidylate synthase ThyX. Here the distinct catalytic reaction directly produces tetrahydrofolate (THF) and consequently in most ThyX-containing organisms, DHFR is absent. While the resulting influence on the folate metabolism of ThyX-containing bacteria is not fully understood, the presence of ThyX may provide growth benefits under conditions where the level of reduced folate derivatives is compromised. Interestingly, the third key enzyme implicated in generation of MTHF, serine hydroxymethyltransferase (SHMT), has a universal phylogenetic distribution, but remains understudied in ThyX-containg bacteria. To obtain functional insight into these ThyX-dependent thymidylate/folate cycles, we characterized the predicted SHMT from the ThyX-containing bacterium *Helicobacter pylori*. Serine hydroxymethyltransferase activity was confirmed by functional genetic complementation of a *glyA*-inactivated *E*. *coli* strain. A *H*. *pylori* Δ*glyA* strain was obtained, but exhibited markedly slowed growth and had lost the virulence factor CagA. Biochemical and spectroscopic evidence indicated formation of a characteristic enzyme-PLP-glycine-folate complex and revealed unexpectedly weak binding affinity of PLP. The three-dimensional structure of the *H*. *pylori* SHMT apoprotein was determined at 2.8Ǻ resolution, suggesting a structural basis for the low affinity of the enzyme for its cofactor. Stabilization of the proposed inactive configuration using small molecules has potential to provide a specific way for inhibiting *Hp*SHMT.

## Introduction

**Fig 1:**
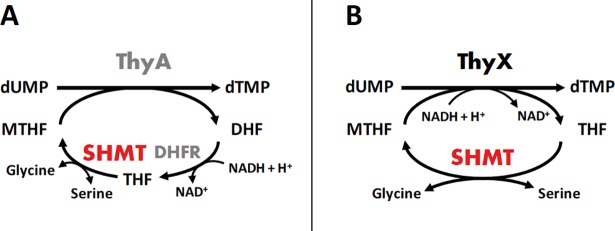
**Thymidylate synthesis cycles involving thymidylate synthase ThyA (A) or thymidylate synthase ThyX (B).** Both enzymes, ThyA and ThyX, perform *de novo* synthesis of deoxythymidine monophosphate (dTMP; thymidylate) from deoxyuridine monophosphate (dUMP). ThyA proteins use methylenetetrahydrofolate (MTHF) both as carbon and electron source, resulting in the formation of dihydrofolate (DHF), which is subsequently reduced by dihydrofolate reductase (DHFR). The ThyX flavoenzymes produce tetrahydrofolate (THF) as reaction product and not dihydrofolate; consequently DHFR is absent. This key difference in ThyA and ThyX catalysis has important implications for the bacterial folate metabolism. The third key enzyme implicated in generation of MTHF, serine hydroxymethyltransferase (SHMT), has a universal phylogenetic distribution.

Serine hydroxymethyltransferase (SHMT or GlyA; EC 2.1.2.1) is a ubiquitous pyridoxal 5’-phosphate (PLP)-dependent enzyme. Its physiologically relevant reaction is the reversible interconversion of serine and tetrahydrofolate (THF) to glycine and 5,10-methylene tetrahydrofolate (MTHF) [1–3]. In addition, SHMT has also been shown to catalyze THF-independent aldolytic cleavage, decarboxylation and transamination reactions [4, 5]. As MTHF is a major source of cellular one-carbon units, SHMT is a pivotal metabolic enzyme for the biosynthesis of purines and thymidylate [6, 7]. Its important role in DNA synthesis, together with the high level of enzyme activity in rapidly proliferating cells, have focused attention on SHMT as a potential target for cancer therapy and for the development of antimicrobial agents [1, 8]. It is becoming increasingly obvious that metabolic adaptation plays a central role in the interaction of bacterial pathogens with their host, supporting the concept of nutritional virulence [9]. Recently, Dahal *et al*. [10] showed that a *glyA* deletion mutant of *Edwardsiella ictaluri* was significantly attenuated in virulence. Bogard *et al*. identified SHMT (named GlyA1 in their study) as a new MetR-regulated virulence factor, required by *V*. *cholerae* to colonize the infant mouse intestine [11]. In mammals, cytoplasmic and mitochondrial isoforms of SHMT are present that form homotetramers of four identical subunits [6], whereas microbial systems contain in general one single homodimeric enzyme [12, 13]. Serine hydroxymethyltransferases, encoded by the *glyA* genes, are structurally highly conserved, with the active site located at the interface between two monomers. In Eukarya and most bacterial species, SHMT is part of the thymidylate/folate cycle, together with canonical thymidylate synthase ThyA (EC 2.1.1.45) and dihydrofolate reductase DHFR (EC 1.5.1.3) [14]. For several decades, the functional association of SHMT, ThyA and DHFR was thought to be a universally conserved evolutionary feature (**Fig 1A**). This view changed with the discovery of a novel family of flavin-dependent thymidylate synthases, ThyX, essential for *de novo* dTMP synthesis (EC 2.1.1.148) [15] (**Fig 1B**). ThyX proteins use distinct reductive and catalytic mechanisms that, differently from ThyA, directly produce THF and not DHF. How the almost universal absence of DHFR in ThyX-containing species [16, 17] influences their folate metabolism is not fully understood. However, on the basis of mathematical modeling of the bacterial folate metabolism, we have proposed that a very low dihydrofolate reductase activity, provided by moonlighting enzymes, is sufficient to rescue thymidylate synthesis in the presence of ThyX. Therefore, the presence of flavin-dependent thymidylate synthase X may provide growth benefits under conditions where the level of reduced folate derivatives is compromised [18]. In contrast, the third key enzyme implicated in generation of MTHF, SHMT, has a universal phylogenetic distribution.

Interestingly, biochemical and genetic studies, supported by statistical analyses of microbial genome compositions, suggested that the catalytic efficiency of ThyX enzymes is relatively low in comparison to ThyA [19]. Based upon *in silico* analyses using the KEGG database [20, 21], in the *thyX*-containing bacterium *H*. *pylori* the only direct way for MTHF synthesis implicates the thymidylate cycle comprising ThyX and SHMT in the absence of DHFR. Similar phylogenetic profiles have been observed in most other ThyX-containing bacteria. The function of other orthologues, potentially implicated in the synthesis of folate derivatives, has not been studied in *Helicobacter pylori* and very few studies have addressed the biochemical properties or the metabolic implication of SHMT in *thyX*-containing species. Thus, despite their importance for pathogenicity, the folate/thymidylate pathways are understudied in bacterial species relying on thymidylate synthase ThyX. In a study investigating the metabolic importance of glycine decarboxylase in cyanobacteria, the presence of *glyA* was found to be essential for cell viability under standard conditions in the strain *Synechocystis* sp. PCC 6803 [22]. In *Mycobacterium tuberculosis* that carries both thymidylate synthases, ThyA and ThyX, two recombinant homodimeric serine hydroxymethyltransferases, named SHM1 and SHM2, with different thermal stabilities and PLP stoichiometries were described. L-cysteine was found to remove PLP from both enzymes, leaving the respective inactive apoenzymes [23]. Serine hydroxymethyltransferase from *Mycobacterium leprae*, also carrying both, ThyA and ThyX, was found to have a low PLP-content and a relatively low catalytic efficiency under the reaction conditions tested [24]. That study also reported that at pH values below 6.0 and above 10.0, PLP was lost from the protein, resulting in the loss of enzymatic activity. Altogether, these findings motivated us to characterize SHMT, the only universally conserved enzyme of the thymidylate metabolic cycle, in the ThyX-containing pathogenic bacterium *H*. *pylori*.

In *H*. *pylori* strain 26695, the open reading frame (ORF) HP0183 was annotated to code for a serine hydroxymethyltransferase. In the present study, the activity of the corresponding polypeptide was confirmed by functional complementation of *E*. *coli* SHMT *in vivo*, supported by biochemical studies. A *H*. *pylori glyA* deletion strain was viable, but exhibited markedly slowed growth compared to wild type and had lost the virulence factor CagA. We solved the three-dimensional structure of the *H*. *pylori* SHMT apoprotein at a resolution of 2.8 Ǻ, which revealed a disordered active site and provided structural insight into the low affinity of the enzyme for its PLP cofactor. Stabilization of the proposed inactive configuration using small molecules may provide a specific way for inhibiting *Hp*SHMT.

## Materials and methods

### Chemicals

Ampicillin, kanamycin, L-allothreonine, alcohol dehydrogenase, pyridoxal 5′-phosphate monohydrate, glycine and serine were purchased from Sigma Aldrich. 5,10-methylenetetrahydrofolate was provided by Merck Eprova AG. Reagents for bacterial and cellular growth were obtained from Gibco, Sigma Aldrich and Oxoïd.

### Molecular techniques

Agarose gel and SDS-PAGE electrophoreses were carried out following standard procedures [25]. All restriction enzymes and GoTaq^®^ Flexi DNA polymerase were purchased from Fermentas-Thermo Fisher Scientific and used according to the manufacturer’s recommendations. Oligonucleotide primers used for PCR amplification were synthesized by Eurogentec and are listed in **Table 1**. Plasmids were extracted and purified using the Qiagen Plasmid Mini Kit. The NucleoSpin Tissue Kit (Macherey-Nagel) was used to extract chromosomal DNA from *H*. *pylori* strains.

**Table 1:** Nucleotide sequences of oligonucleotides used in this study.

**Name**	**Target gene or locus**	**Sequence (5’ to 3’)**^*a*^^,^^*b*^
**SA36**	*glyA / HP0183 (H*. *pylori)*	GAggattcATGGCGTATTTTTTAGAACAAACG
**SA37**		CTTGagatctTTAAAAAATAGGTTGGTGGTACACAG
**oEF22**		GAGGCTATGGGGAGTGTTTT
**oEF23**		CGCCATAAGAAAAGCTCTGA
**SK40**	*HP0182 (H*. *pylori)*	TCGCTAACGGCTTTAGCGAGT
**SK41**	*HP0184 (H*. *pylori)*	GGCGTTTTGGTGTTATAAGCG
**SA80**	*cagA / HP0547 (H*. *pylori)*	AGTGGTTTGGGTGGTGTAGG
**SA81**		CGTAAATGGGTTCAGGGCTA
**SA82**		TGGCGTTTCCCATTTAGAAG
**SA83**		CTCCAAATGCTCTCGTTTCC
**SA129**		ATGACTAACGAAACTATTGATCAAAC
**SA130**		TTAAGATTTTTGGAAACCACCTTTTG
**SA39**	*glyA (E*. *coli)* upstream region	GGGCTTCACGTTGATCGCCATTACGCTGGTTAC
**SA41**	*glyA (E*. *coli)* downstream region and *aphA-3* cassette	TACCTGGAGGGAATAATGGCGAAACGGTGATTTGCTGTC
**SA42**	*glyA (E*. *coli)* upstream region	GTTAATCGCTGCCTGGCAAAGTGGAGAACC
**SA43**	*glyA (E*. *coli)* upstream region and *aphA-3* cassette	GTTAGTCACCCGGGTACCCGCATCTCCTGACTCAGCTA
**SA44**	*glyA (E*. *coli)* upstream region	GCCTCGCGATTGATAAATACA
**SA45**	*glyA (E*. *coli)* downstream region	GTCTGCGACTGTGGACGTTAT
**H50**	*aphA-3 cassette*	CCGGTGATATTCTCATTTTAGCC
**H17**		TTTGACTTACTGGGGATCAAGCCTG

^*a*^ Lowercase letters indicate *Bam*HI (SA36) and *BgI*II (SA37) restriction sites.

^*b*^ Underlined sequences correspond to homologous nucleotides of the *aphA-3* cassette

### Bacterial strains

*E*. *coli* strain SURE (*e14*^*–*^ *(McrA*^*–*^*) Δ(mcrCB-hsdSMR-mrr)1*71 *endA1 supE44 thi-1 gyrA96 relA1 lac recB recJ sbcC umuC*::*Tn5* (Kan^r^) *uvrC* [F' *proAB lacI*^*q*^*ZD(M15 Tn10* (Tet^r^)]) was used as host for plasmid cloning experiments. *E*. *coli* strain SG 13009, containing pREP4 [26], was used for expression of recombinant *Hp*SHMT cloned into the pQE60 expression plasmid (Qiagen). *E*. *coli* MG1665 (F- lambda- *ilvG*- *rfb*-50 *rph*-1) was used for deletion of *glyA*. All *E*. *coli* strains were grown at 37°C on solid or in liquid LB medium or M9 minimal medium (3 g L^-1^ Na_2_HPO_4_, 1.5 g L^-1^ KH_2_PO_4_, 0.25 g L^-1^ NH_4_Cl, and 0.15 g L^-1^ NaCl) supplemented with 2 mM MgSO_4_, 0.1 mM CaCl_2_ and 0.1% glycerol as carbon source. Antibiotics for the selection of recombinant *E*. *coli* strains were used at the following final concentrations: ampicillin, 100 μg ml^−1^; kanamycin, 50 μg ml^−1^ and tetracycline 5 μg ml^−1^. *H*. *pylori* strain 26695 (NC_000915.1) [27] was used as recipient for isogenic mutant construction and was cultured on horse blood (10%) agar or in brain–heart infusion (BHI) (Oxoid) liquid medium supplemented with fetal calf serum (10%) and with antibiotic and fungicide mix consisting of vancomycin (final concentration, 10 μg ml^−1^), polymyxin B (2.5 IU l^−1^), trimethoprim (5 μg ml^−1^), amphotericin B (4 μg ml^−1^) and fungizone (2.5 μg ml^−1^). Plates and flasks were incubated for 48 h at 37°C under microaerobic conditions [28]. For mutant strains derived from *H*. *pylori* strain 26695 by allelic exchange, kanamycin was added to the growth medium at a final concentration of 20 μg ml^−1^. *H*. *pylori* strain X47, a *cagA*-deficient strain, was used as control in immunoblot experiments.

### Cloning, expression and purification of *H*. *pylori* SHMT

Plasmid pQE60 (Qiagen), carrying the *glyA* gene (HP0183; NP_206982.1) from *H*. *pylori* strain 26695, contains an IPTG-inducible *tac* promoter and an ampicillin selection marker. The *glyA* gene was PCR-amplified using primer pair SA36/SA37 (**Table 1**) and inserted into unique *Bam*HI and *Bgl*II sites. This construct, carrying a hexahistidine (6xHis)-tag at its carboxyl terminus, was confirmed by DNA sequencing. The *Hp*SHMT recombinant protein was expressed in *E*. *coli* SG 13009 at 37°C in 750 ml LB medium. Protein expression was induced by adding 1 mM IPTG to early exponential phase cultures (OD_600_ 0.5) for 3 h. 6xHis-tagged proteins were purified from cell-free extracts by gravity-flow chromatography on Ni-TED columns (Macherey Nagel), followed by subsequent imidazole removal on Econo-Pac columns (Bio-Rad). The eluted protein was stored at -80°C. Protein samples were analyzed on 10% SDS-PAGE and by quantitative capillary electrophoresis (Experion, Bio-Rad) and were more than 95% pure.

### Construction of *glyA* mutants

#### Deletion of *glyA* in *E*. *coli*

The *EcΔglyA* mutant was constructed in *E*. *coli* strain MG1655 using a three-step PCR procedure as described in [29]. First, the 492 bp sequence located upstream of *glyA* (b2551; NP_417046.1) and the 513 bp sequence located downstream of *glyA* were PCR-amplified with primer pairs SA39/SA43 and SA41/SA42, respectively (**Table 1**). The SA43 and SA41 oligonucleotides have 21 and 18 bp homology to the upstream and downstream flanking regions of *glyA*, followed by 17 and 21 bp homology to the 5’ and 3’ region of the *aphA-3* kanamycin cassette, respectively. The non-polar cassette was obtained by *Sma*I digestion of pUC18K [30, 31]. The deletion construct was assembled by two sequential PCRs and then introduced into the chromosome of *E*. *coli* using the Lambda Red recombination system. To this end, MG1655 was previously transformed with the thermosensitive pKOBEGA plasmid (kindly provided by J-M. Ghigo, Pasteur Institute, France) that carries the λ phage *redγβα* operon under the control of a pBAD promoter [29]. Recombinants were selected with kanamycin at the non-permissive temperature of 37°C (permissive temperature = 30°C) and were tested for chloramphenicol sensitivity to check for loss of pKOBEGA. Correct allelic exchange was confirmed by PCR.

#### Deletion of *glyA* in *H*. *pylori*

Plasmid pILL570 (*glyA*::*aphA-*3) (kindly provided by H. de Reuse, Pasteur Institute, France) is a derivative of the pILL570 vector [32], in which the non-polar *aphA-3* kanamycin resistance cassette was inserted into a unique site generated after cloning of a 318-bp fragment, corresponding to the HP0183 5′ end, and a 304-bp fragment, corresponding to the HP0183 3′ end, respectively. The *H*. *pylori* mutants were obtained by natural transformation [33] with approximately 2 μg of plasmid DNA. Clones that had undergone allelic exchange were selected after seven days of growth on plates containing 20 μg ml^−1^ kanamycin. Correct allelic exchange was confirmed by PCR (**S1 Fig**).

### Genome sequencing

To sequence the *H*. *pylori ΔglyA* mutant strain, library preparation was carried out using the Ion Xpress^™^ Plus Fragment Library Kit (Life Technologies, Thermo Fisher Scientific), with 315 ng of genomic DNA (gDNA). gDNA was enzymatically fragmented using the Ion Shear^™^ Plus method. Adapter ligation, size selection, nick repair and amplification were performed for 1 μg of gDNA, as described in the Ion Torrent protocol furnished with the kit. Treated gDNA was subsequently loaded on an E-Gel^®^ SizeSelect^™^ 2% agarose gel and fragments with sizes ranging from 450 to 480 bp were extracted. Amplification and enrichment steps were carried out using the Ion PGM^™^ Template OT2 400 Kit. The Ion PGM^™^ Template OT2 400 Ion Sphere^™^ particles’ quality was assessed with the Ion Sphere^™^ Control Kit and a Qubit 2.0 fluorometer (Life Technologies-Thermo Fisher Scientific). Sequencing was performed using a 314^™^ Chip v2 and the Ion PGM^™^ Sequencing 400 Kit. Once loaded, the chip was placed on the Ion PGM^™^ Sequencer. Coverage analysis reports (**S1 Table and S2 Fig**) were generated using a coverage analysis plugin (v5.8.0.8) of the IonTorrent server, indicating average base coverage depths of 32.06 and 76.78 for wild type and *ΔglyA H*. *pylori* strains, respectively. Where indicated, sequencing reads were mapped to the *H*. *pylori* 26695 reference genome (NC_000915.1) using Artemis software [34].

### Biochemical analyses

#### Ternary complex formation

*E*. *coli* SHMT forms a PLP-dependent ternary complex with glycine and MTHF that absorbs at 502 nm. The affinity of *H*. *pylori* SHMT for MTHF was determined by measuring the absorbance at 502 nm as a function of the MTHF concentration at fixed concentrations of glycine [35]. Reactions were performed at room temperature in 50 mM phosphate buffer, pH 8.0, in the presence of 150 mM NaCl, 1 mM EDTA, 1 mM DTT, 20 μM SHMT, 250 μM PLP, 5 mM glycine and 0–2 mM MTHF.

#### Activity measurements

The activity of SHMT was measured indirectly using L-allothreonine as substrate. In this reaction, SHMT catalyzes the cleavage of L-allothreonine to glycine and acetaldehyde. The produced acetaldehyde is reduced to ethanol by the NADH-dependent alcohol dehydrogenase and the oxidation of NADH to NAD^+^ was monitored at 340 nm [36]. Reactions were performed at room temperature, in 50 mM phosphate buffer, pH 8.0, in the presence of 150 mM NaCl, 1 mM EDTA, 1 mM DTT, 20 μM SHMT, 250 μM PLP, 20 μg alcohol dehydrogenase and 250 μM NADH. NADH consumed in the reaction was calculated using a molar extinction coefficient of 6220 M^-1^cm^-1^ [37].

#### Immunoblot analyses

*H*. *pylori* cells were harvested by centrifugation at 4200 g for 5 min. *H*. *pylori* proteins were separated on SDS PAGE and stained with Coomassie Brilliant Blue R250 or were used for immunoblot analysis. Proteins were transferred at 4°C to a nitrocellulose membrane (GE Healthcare) using a Transblot apparatus (Bio-Rad). Following transfer, membranes were blocked in Odyssey Blocking Buffer (Li-Cor Biosciences) for one hour. The membranes were incubated with primary antibody (diluted 1:1000) for one hour or overnight, washed four times for 5 minutes each at room temperature in PBS + 0.1% Tween-20, and subsequently incubated for one hour at room temperature with the fluorescently-labeled secondary antibody (diluted 1:1000 in Odyssey Blocking Buffer). Mouse monoclonal anti-polyhistidine antibody (Sigma-Aldrich), rabbit polyclonal anti-UreB antibody (Abcam), and mouse monoclonal anti-*H*. *pylori* CagA antibody (Abnova) were used as primary antibodies. IRDye 800CW conjugated goat polyclonal anti-rabbit and anti-Mouse IgG were used as secondary antibodies (Li-Cor Biosciences). The membranes were washed four times for 5 minutes each at room temperature in PBS + 0.1% Tween-20 with gentle shaking protected from light. The membrane was scanned using the Odyssey Infrared Imaging system following manufacturer’s instructions.

### Crystallisation, data collection, structure determination and refinement

Crystals of *Hp*SHMT were grown using the vapour diffusion method with hanging drops consisting of 1μl of protein (7 mg ml^-1^) and 1μl of reservoir solution (20% PEG 3350, 0.2 M sodium acetate). Small (10μm x 10μm x 50μm) needle crystals grew after a few days at room temperature. For data collection, the crystal was transferred into silicon oil and subsequently flash frozen in liquid nitrogen. Data were collected at 100 K at the European Synchrotron Radiation Facility (ESRF) beamline ID14EH4. Diffraction images were integrated and scaled with X-ray Detector Software (XDS) [38], then symmetry-related intensities were merged and structure factors derived using Aimless from CCP4 Program Suite [39]. *Hp*SHMT crystals belonged to the space group P2_1_2_1_2_1_ and diffracted anisotropically. In particular, the CC_1/2_ of b* was 0.056 at the highest resolution shell, with an I/σI of 0.34. After several different processing experiments we decided to use the overall resolution of 2.8 Å. Exploiting higher resolution data did not change significantly either the structure or our conclusions. The structure was solved by molecular replacement (MR) using a homology model of *Hp*SHMT generated with the Swiss Modelling server [40] that used the *Thermus thermophilus* SHMT (*Tt*SHMT) structure as template (PDB ID 2DKJ) and the program PHASER [41]. PHENIX [41] was used for refinement, alternated with manual model building in COOT [42]. Residues 4–411 were modelled for chain A and residues 3–416 for chain B, with the exception of several loops where the path of the peptide backbone could not be traced. Hence, residues 53–66 and 124–130 were omitted in chain A and residues 54–67 and 120–134 were absent in chain B. Data collection and refinement statistics of the final model are presented in **Table 2**. Figures were generated with Pymol Software [43].

**Table 2:** Data collection and refinement statistics for the SHMT structure from *H*. *pylori* 26695.

***Data collection***			
Beamline		ID14eh4-ESRF	
Wavelength (Å)		0.9790	
Space group		*P*2_1_2_1_2_1_	
Unit cell dimensions (Å)		*a* = 57.349 *b* = 87.548 c = 162.476 α = β = *γ* = 90	
Resolution range (Å)		46.85–2.80 (2.95–2.85)	
Unique reflections		20625 (2920)	
*R*_sym_ (%)		11.7 (54.6)	
R_pim_ (%)		11.3 (52.5)	
CC1/2		0.999 (0.925)	
CC*		0.994 (0.749)	
Completeness (%)		99.1 (98.7)	
Multiplicity		3.6 (3.6)	
<I/s(I)>		10.2 (2.3)	
***Refined model composition***			
Monomers / a. u		2	
Protein residues			
Molecule A		A4-A52	
		A67-A123	
		A131-A411	
Molecule B		B3-B53	
		B68-B119	
		B135-B416	
Water molecules		16	
Wilson *B*-value (Å^2^)	Mean B-Value (Å^2^)	42.01	43.1
***Model quality indicators***			
*R*_work_ / *R*_free_ (%)		19.53/23.56	
Rmsd bond lengths (Å)		0.005	
Rmsd bond angles (°)		0.795	
Estimated coordinate error (Å)		0.37	
Molprobity clash/overall scores		2.26/9.6	
^***b***^***Ramachandran analysis***			
% Favoured	% Allowed	95.29	4.45
% Disallowed	% Rotamer outlier	0.26	3.76
**PDB ID code**		6F93	

**Accession number.** The coordinates and structure factors of *Hp*SHMT were deposited in the Protein Data Bank with the entry code 6F93 (PDB ID 6F93).

## Results

### HP0183 complements an *E*. *coli* Δ*glyA* strain *in vivo*

**Fig 2:**
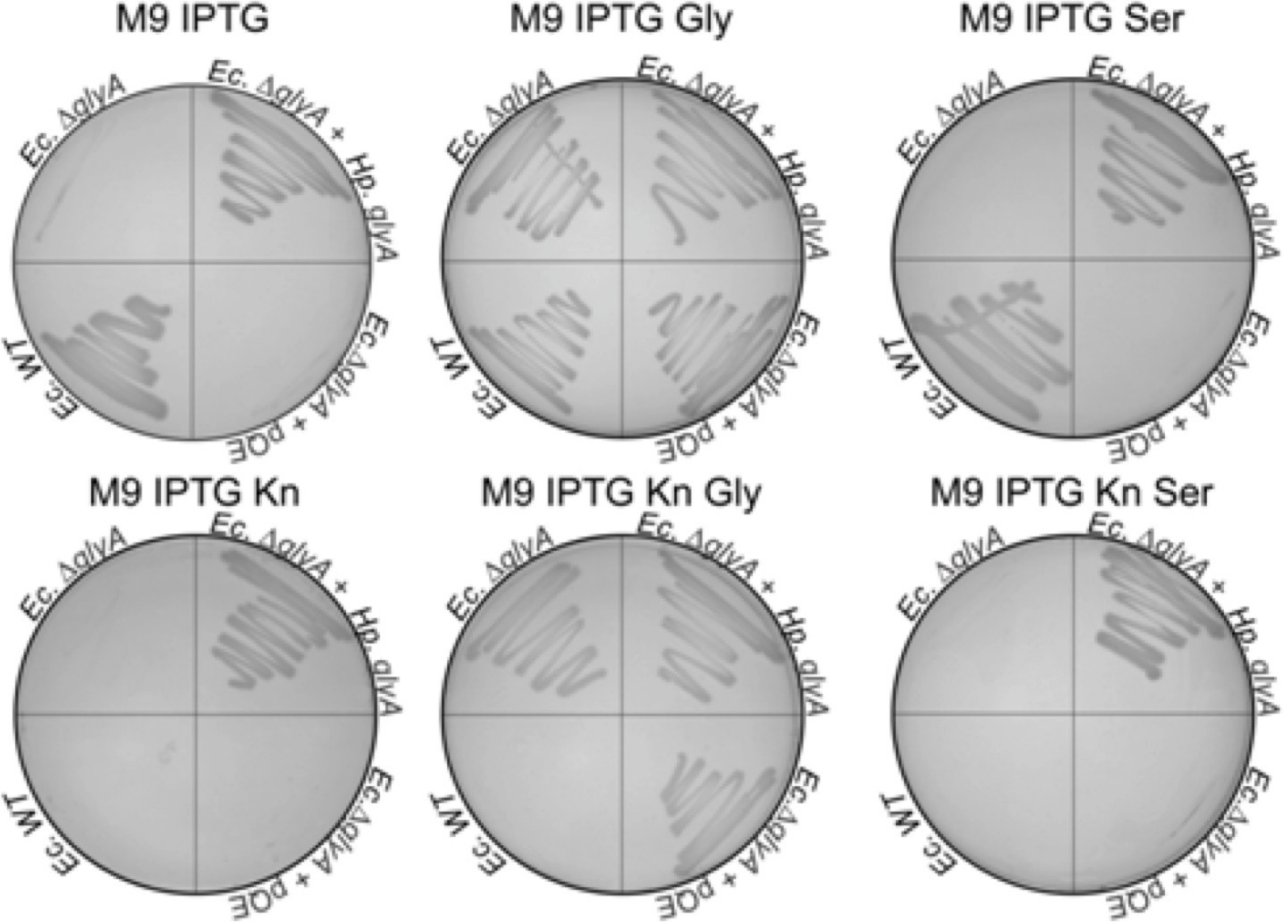
Genetic complementation assays *in vivo*, testing for the function of HP0183. The *glyA* gene in *E*. *coli* strain MG1665 was partially deleted and replaced with a kanamycin (Kn) resistance cassette. The resulting deletion strain *Ec*Δ*glyA* was transformed with plasmid pQE60, containing the HP0183 gene under control of an IPTG-inducible T5 promoter, resulting in strain *Ec*Δ*glyA + HpglyA*. Complementation tests were performed on solid M9 minimal medium in the presence of glycine (Gly) and serine (Ser). WT *E*. *coli* MG1655 was used as positive control, *Ec****Δ****glyA* transformed with pQE60 without insert (*Ec****Δ****glyA* + pQE) as negative control.

In *H*. *pylori* 26695, the open reading frame HP0183 (1248 bp) is located at chromosomal positions 190186–191436. This ORF is part of a multigene operon where two non-essential ORFs (HP0184 and HP0185) are located downstream from HP0183 (http://csbl.bmb.uga.edu/DOOR/operon.php?id=3975). As the translated polypeptide shows (e.g.) 53% sequence identity (72% sequence similarity) with *E*. *coli* SHMT (*Ec*SHMT) and 68% sequence identity (80% sequence similarity) with the *Campylobacter jejuni* enzyme, ORF HP0183 was predicted to code for a serine hydroxymethyltransferase. Moreover, BLAST searches using default parameters revealed the presence of *glyA* genes in more than 500 sequenced *H*. *pylori* strains. As these hits were 96–100% identical with HP0183 of *H*. *pylori* 26695, these findings indicate that this gene is highly conserved in a wide range of clinical isolates. It has been shown for *E*. *coli* [44, 45] and humans [46] that SHMT-deficiency induces glycine-auxotrophy. This reflects the presence of a glycine-inducible glycine cleavage system that provides an alternative biosynthetic route for MTHF. As there is no *in silico* evidence for the presence of a glycine cleavage system in *H*. *pylori*, we tested directly, whether the polypeptide encoded by HP0183 can functionally complement growth defects of an *E*. *coli* strain specifically impaired in serine hydroxymethyltransferase activity. To this effect we interrupted the *glyA* gene in the *E*. *coli* strain MG1665 with a kanamycin resistance cassette (see Materials and methods). We found the *E*. *coli ΔglyA* deletion strain to be glycine-auxotroph in agreement with earlier results. The *E*. *coli ΔglyA* strain was subsequently transformed with plasmid pQE60 carrying the IPTG-inducible ORF HP0183, and functional complementation tests on solid minimal M9 medium were performed in the presence or absence of glycine and serine. WT *E*. *coli* MG1665 and the deletion strain transformed with pQE60 without insert served as controls. In the presence of IPTG, transformants carrying the ORF HP0183 on the plasmid were found to be capable of growth on minimal medium, as does the WT *lacI*^+^ *E*. *coli* strain MG1665, containing the functional *glyA* gene (**Fig 2**). This genetic complementation test confirms as well that the *E*. *coli ΔglyA* strain was not able to grow on minimal medium supplemented with serine, supporting the hypothesis that under these conditions in *E*. *coli* SHMT catalyses in priority the conversion of serine to glycine and not the inverse reaction.

### Cloning, expression, purification and activity of *Helicobacter pylori* SHMT

**Fig 3:**
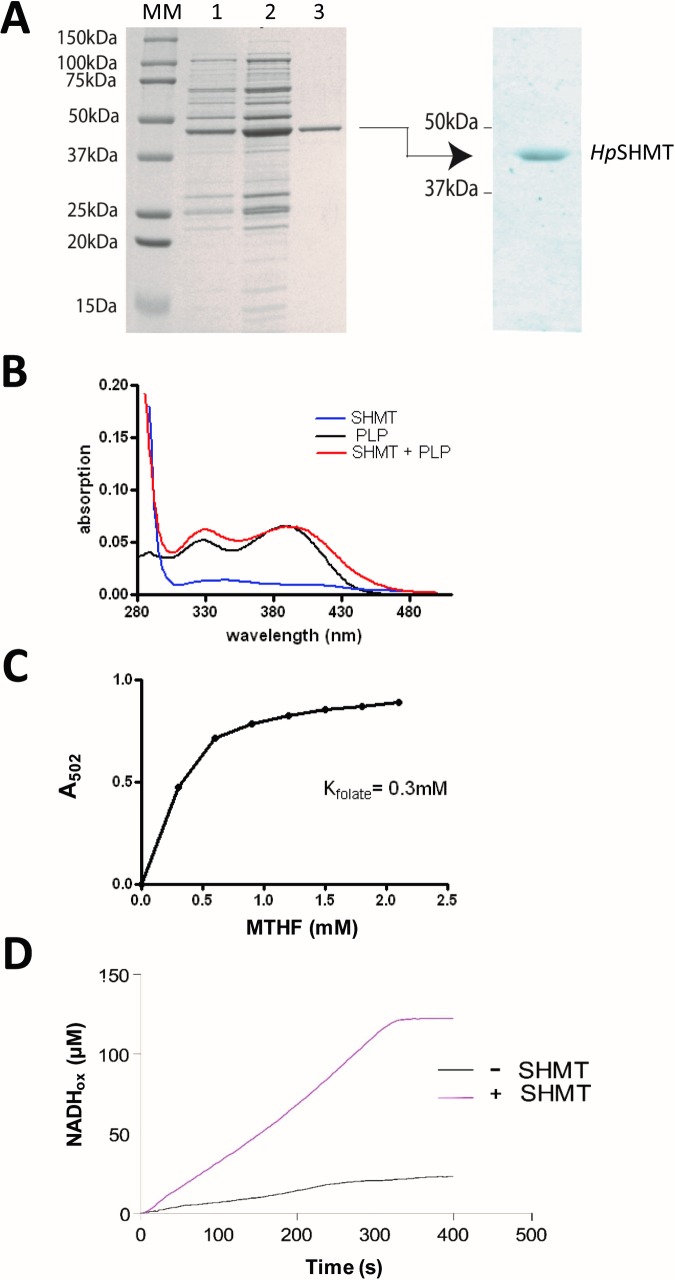
Biochemical properties of *Hp*SHMT. **(A)** Expression and purification of *Hp*SHMT. *Left*: SDS-PAGE (15%) analysis. Lane 1: crude extracts before IPTG induction; lane 2: crude extracts 3h after IPTG induction; lane 3: purified *Hp*SHMT; MM: molecular weight standards. The purified protein (2 μg) shows an apparent molecular mass of ≈ 45 kDa. *Right*: immunodetection of *Hp*SHMT with monoclonal anti-6xHis antibody. **(B)** Absorption spectrum of purified *Hp*SHMT (blue curve), free PLP in solution (black curve) and a stoichiometric mixture *Hp*SHMT/PLP (monomer : PLP = 1 : 1) (red curve) registered at room temperature. The latter presents a characteristic shift of the peak to 421 nm, corresponding to the Schiff base formed between the pyridoxal cofactor and an amino group of the protein. **(C)** Measurement of the affinity of *Hp*SHMT for MTHF based upon the absorption at 502 nm, corresponding to the intermediate ternary enzyme-PLP-glycine-folate complex, using increasing substrate concentrations. An apparent binding constant was determined by plotting the absorption values at 502 nm as a function of the MTHF-concentration. The values shown are averages of two independent measurements that were within 20% of each other. **(D)** Oxidation of NADH and *Hp*SHMT activity. SHMT also catalyzes the folate-independent retroaldol cleavage of allothreonine and 3-phenylserine to glycine and acetaldehyde. The reduction of acetaldehyde to ethanol via an NADH-dependent alcohol dehydrogenase directly links the oxidation of NADH to the enzymatic activity of SHMT. Red curve: with *Hp*SHMT; grey curve: without *Hp*SHMT.

The polypeptide corresponding to HP0183 was expressed in *E*. *coli* SG 13009 (containing pREP4 that carries *lacI*^*q*^); see Materials and methods) with a C-terminal 6xHistidine-tag and purified using Ni-affinity chromatography followed by gel filtration chromatography. The purified protein (purity >95%) shows an apparent molecular mass of ≈ 45 kDa on SDS PAGE (predicted molecular mass per monomer 46,776 Da) and is recognized by immunodetection with monoclonal anti-6xHis antibodies **(Fig 3A).** Dynamic light scattering measurements characterized *Hp*SHMT as a polydispersed sample with the majority of particles showing a size of approximately 10 nm, indicative for a dimeric state of the protein. A small fraction of purified *Hp*SHMT had a particle size of about 100 nm, likely representing aggregation products.

The purified *H*. *pylori* SHMT protein exhibits a very faint yellow colour, suggesting that an expected pyridoxal 5’-phosphate cofactor might be present only at small concentrations. The absorption spectra of the purified enzyme and of free PLP were measured between 280 and 500 nm. Free PLP in solution shows two distinct absorption peaks at 327 and 390 nm. The absorption spectrum of the enzyme and PLP in stoichiometric quantities (monomer : PLP = 1 : 1) shows a shift of the 390 nm peak to 421 nm, corresponding to the formation of a Schiff base between the aldehyde group of PLP and an amino (NH_2_) group of the protein, typical for SHMT enzymes **(Fig 3B).** These findings indicate that, differently from most other serine hydroxymethyltransferases, *Hp*SHMT binds its cofactor PLP only weakly, essentially resulting in the loss of PLP during protein purification.

Serine hydroxymethyltransferases are capable of catalysing both, folate-dependent and folate-independent reactions, with the former being the main physiological reaction, involving the reversible interconversion of serine to glycine. The affinity of SHMT for MTHF can be determined by measuring the absorbance at 502 nm, corresponding to the intermediate ternary enzyme-PLP-glycine-folate complex, with increasing substrate concentrations [35]. An apparent binding constant for MTHF was determined by plotting the absorption values at 502 nm as a function of the MTHF-concentration (**Fig 3C**). A concentration of 0.3 mM of folate substrate resulted in 50% of the maximal signal. This value was considered as apparent K_Fol_ value and is similar to what is observed for the *E*. *coli* enzyme (K_Fol_, 0.1 mM) [35] and cytoplasmic (K_Fol_, 0.2 mM) or mitochondrial (K_Fol_, 0.07 mM) SHMTs from rabbit under similar reaction conditions [47, 48]. Our data indicate that the *H*. *pylori* enzyme forms a stable ternary complex upon external addition of pyridoxal 5’-phosphate, characteristic for SHMTs.

In addition, SHMT is able to catalyze the folate-independent retroaldol cleavage of L-allothreonine and 3-phenylserine to glycine and acetaldehyde [36]. The produced acetaldehyde is reduced to ethanol by NADH-dependent alcohol dehydrogenase, and the oxidation of NADH to NAD^+^ can be monitored at 340 nm, thus establishing a direct link between NADH oxidation and the catalytic activity of SHMT. The measured oxidation curve for the *H*. *pylori* enzyme is presented in **Fig 3D** and indicates that it is able to transform L-allothreonine to acetaldehyde, thus confirming its activity as SHMT.

### Structure of SHMT from *Helicobacter pylori*

**Fig 4:**
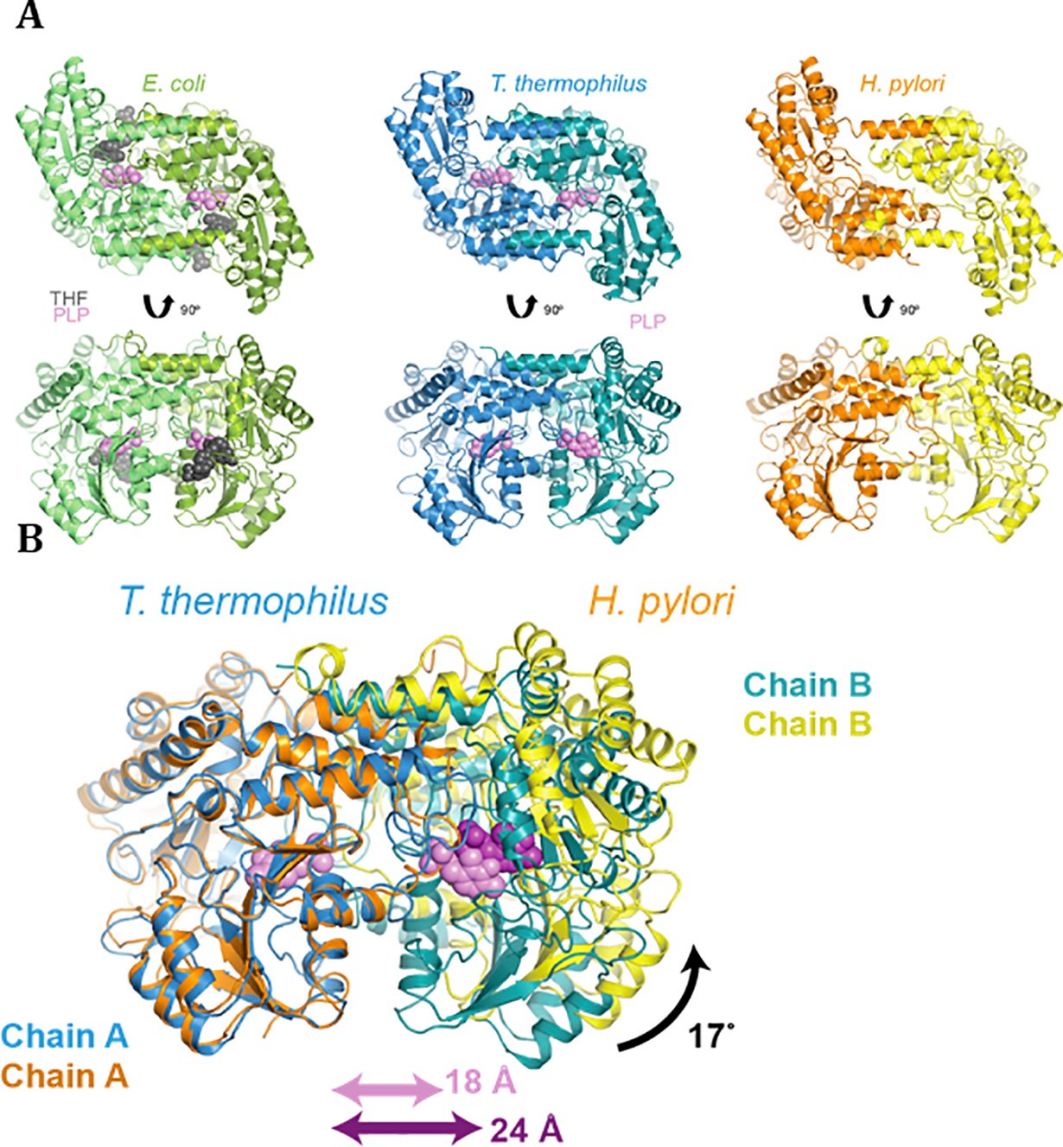
Structural characteristics of *Hp*SHMT. **(A)** Ribbon representations of the dimeric SHMTs from *E*. *coli* (PDB ID 1DFO, [49]), *Thermus thermophilus* (PDB ID 2DKJ) and *H*. *pylori* (PDB ID 6F93). Two representations of each structure are depicted. The position of the PLP cofactor is indicated in the *E*. *coli* and *T*. *thermophilus* enzymes; SHMT of *H*. *pylori* contains no bound PLP in the crystal structure. **(B)** Structural alignment of both *Tt*SHMT and *Hp*SHMT dimers revealing a significant change at the homodimer interface.

Crystal structures of serine hydroxmethyltransferases were determined from prokaryotes [49–54], protozoans [55] and eukaryotes [56–58] and were found to display a characteristic fold, similar to other type I PLP enzymes [59]. Prokaryotic SHMTs are homodimers, whereas the eukaryotic enzymes are homotetramers, with no apparent differences in activity [6]. The active site is located at the dimer interface and delineated by amino acid residues from both dimer subunits. In order to obtain structural and functional insight into the *H*. *pylori* enzyme and address the weak PLP binding observed in purified *Hp*SHMT, we solved the crystal structure of *H*. *pylori* SHMT at a resolution of 2.8 Å (see Material and methods and **Table 2**). *Hp*SHMT crystallized as dimer, like the other known bacterial SHMTs, and no evidence was found for higher oligomeric states in the crystal packing. The *Hp*SHMT structure displays the typical highly conserved overall fold of type I PLP-dependent proteins and is formed by one large and one small domain. The spatial organization is very similar to that observed in *Tt*SHMT and *Ec*SHMT (**Fig 4A**) [49]. The large domain contains a seven-stranded β-sheet surrounded by *α*-helices. All strands, but one, are parallel. The small domain at the C-terminal part contains three short antiparallel strands and four *α*-helices and consists of residues 277–409. The first twenty-five residues of the N-terminus and the last ten residues of the C-terminus interact with the adjacent subunit. Superposition of chains A and B shows the same structure (rmsd of 0.56 Å on 376 Cα), with one loop being slightly more ordered in chain A (**Fig 4A, right**). The structure of *Ec*SHMT (PDB ID 1DFO) was solved in the presence of PLP-glycine and 5-formyl tetrahydropteroylglutamate ([49]; **Fig 4A, left**), while the *Tt*SHMT structure (PDB ID 2DKJ) has PLP covalently linked to a conserved lysine residue (Lys 226 in *Tt*SHMT); **Fig 4A, center**). The active sites (one per monomer) of SHMTs reside at the dimer interface (**Fig 4B**) and amino acids from each subunit are involved in PLP binding.

**Fig 5:**
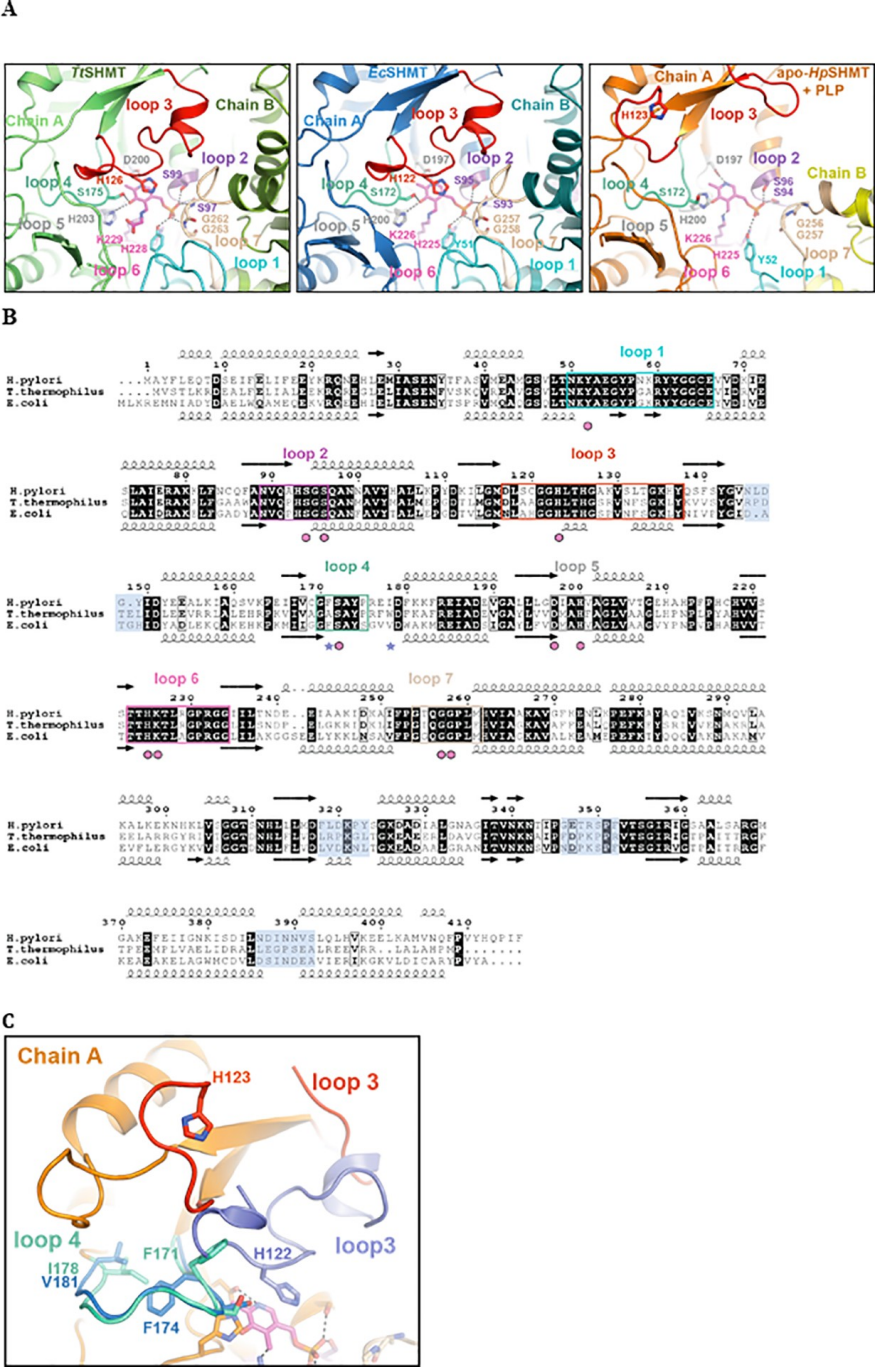
Close-up views of PLP-binding regions and structure-based alignements of SHMTs. **(A)** Close-up view of the PLP-binding region of *Tt*SHMT (left panel), *Ec*SHMT (middle panel) and of a model of *Hp*SHMT apoprotein with PLP (right panel). Contributions of chains A and B and the positions of loops 1, 2, 3, 4, 5, 6 and 7 are indicated in cyan, purple, red, pale green, grey, pink and wheat, respectively. The following residues are missing in loop 1 and 3: 54–67 (53–66) and 124–130 (120–134) from *Hp*SHMT monomer A (monomer B). **(B)** Structure-based alignments of the protein sequences of SHMTs from *E*. *coli*, *T*. *thermophilus* and *H*. *pylori* highlighting structural elements, conserved features (dots), conserved loops 1–7 and residues acting on loop 3 conformation (stars). **(C)** Close-up of the “open lid” conformation of loop 3 from *Hp*SHMT colored in red versus the “closed lid” conformation from *Ec*SHMT colored in blue. Loop 5 is colored in blue and pale green for *Ec*SHMT and *Hp*SHMT, respectively.

The PLP binding pocket is essentially formed by five loops from one subunit, including loop 2 (residues 92–96), loop 3 (residues 118–138), loop 4 (residues 170–174), loop 5 (residues 197–200) and loop 6 (residues 223–227), and is completed by two loops from the adjacent subunit, loop 1 (50–67) and loop 7 (residues 252–259) (**Fig 5A and 5B**). Despite sequence conservation of these seven loops, no PLP was present in the *Hp*SHMT structure, providing one of the very few apoprotein structures of an SHMT enzyme [52, 53]. Superposition of one PLP-bound monomer of *Tt*SHMT on each *Hp*SHMT subunit (rmsd of 1.29Å on 364 Cα) was used to visualise the position of PLP in *Hp*SHMT. The two active sites of *Hp*SHMT are separated by a distance of 24 Å (calculated between the two PLP α-phosphates), which is considerably longer than the average distance of 18 Å observed in most SHMT structures (**Fig 4B**) [60]. This illustrates a modification of the *Hp*SHMT dimer interface, where one subunit is rotated about 17° from the other in comparison to PLP-bound *Tt*SHMT or *Ec*SHMT (**Figs 4B and 5A**). This modification is accompanied by marked differences at the *Hp*SHMT active site loops 1, 3 and 7 (**Fig 5A**). Loop 1 of *Ec*SHMT is essential in both, formation of the homodimer interface and tetrahydrofolate substrate binding, together with loop 3 and a conserved C-terminal loop 429-(residues 344–363) from the adjacent subunit (**Fig 5A**). Remarkably, loop 1 is completely disordered in the two chains of *Hp*SHMT. In the structure of *Ec*SHMT, loop 3 acts as a “lid” for the cofactor and as a “side wall” for the tetrahydrofolate substrate. A conserved histidine residue (His126 in *Tt*SHMT; His122 in *Ec*SHMT) is involved in stacking with PLP in *Tt*SHMT and *Ec*SHMT, and is oriented towards the empty PLP binding site cavity in *Hp*SHMT (**Fig 5A**). In addition, the inter-domain loop 7 closes the PLP binding site in concert with loop 3 from the facing subunit in *Tt*SHMT and *Ec*SHMT (**Fig 5A**). In *Hp*SHMT, loop 7 presents high B-factor values suggesting an increased flexibility (**Fig 4C and Table 2**). Surprisingly, the PLP pocket is found almost pre-organised in one subunit of the *Hp*SHMT structure despite the disorder observed for loops 1, 3 and 7. Indeed, the important residues Y52, S94, S96, S172, D197, H200, G256, G257 from loop 2, 4, 5 and 7 of each subunit are positioned to interact with a PLP moiety **(Fig 5A and 5B)**. This also includes the TTHKTL motif (residues 223–228) containing the Lys 226 residue that covalently binds the 4’ aldehyde of PLP, resulting in the formation of an internal aldimine intermediate, required for SHMT activity.

Interestingly, some residues of *Hp*SHMT could favour an “open lid” conformation of loop 3. *Hp*SHMT Ile 178 (Val in *Ec*SHMT) does not favour the same conformer of Phe 171 in *Ec*SHMT, which in turn is not compatible with a “closed lid” conformation of loop 3 as observed in *Ec*SHMT (**Fig 5C**). It was previously shown that SHMTs could bind either PLP or tetrahydrofolate substrates independently [61]. In this context, binding of the tetrahydrofolate moiety in *Hp*SHMT would likely modify the homodimer interface to bridge loop 1 and the required “closed lid” conformer of loop 3, therefore together promoting PLP binding. To date, most 3D structures of SHMTs were solved with the pyridoxal-phosphate cofactor covalently linked to a conserved lysine residue of the active site, whereas PLP is absent in the structure of *Hp*SHMT reported here. This is in agreement with the low PLP occupancy in purified enzyme preparations, also suggesting that PLP does not bind as strongly to the *H*. *pylori* enzyme as it does in most other SHMTs.

### Functional implication of *Hp*SHMT

**Fig 6:**
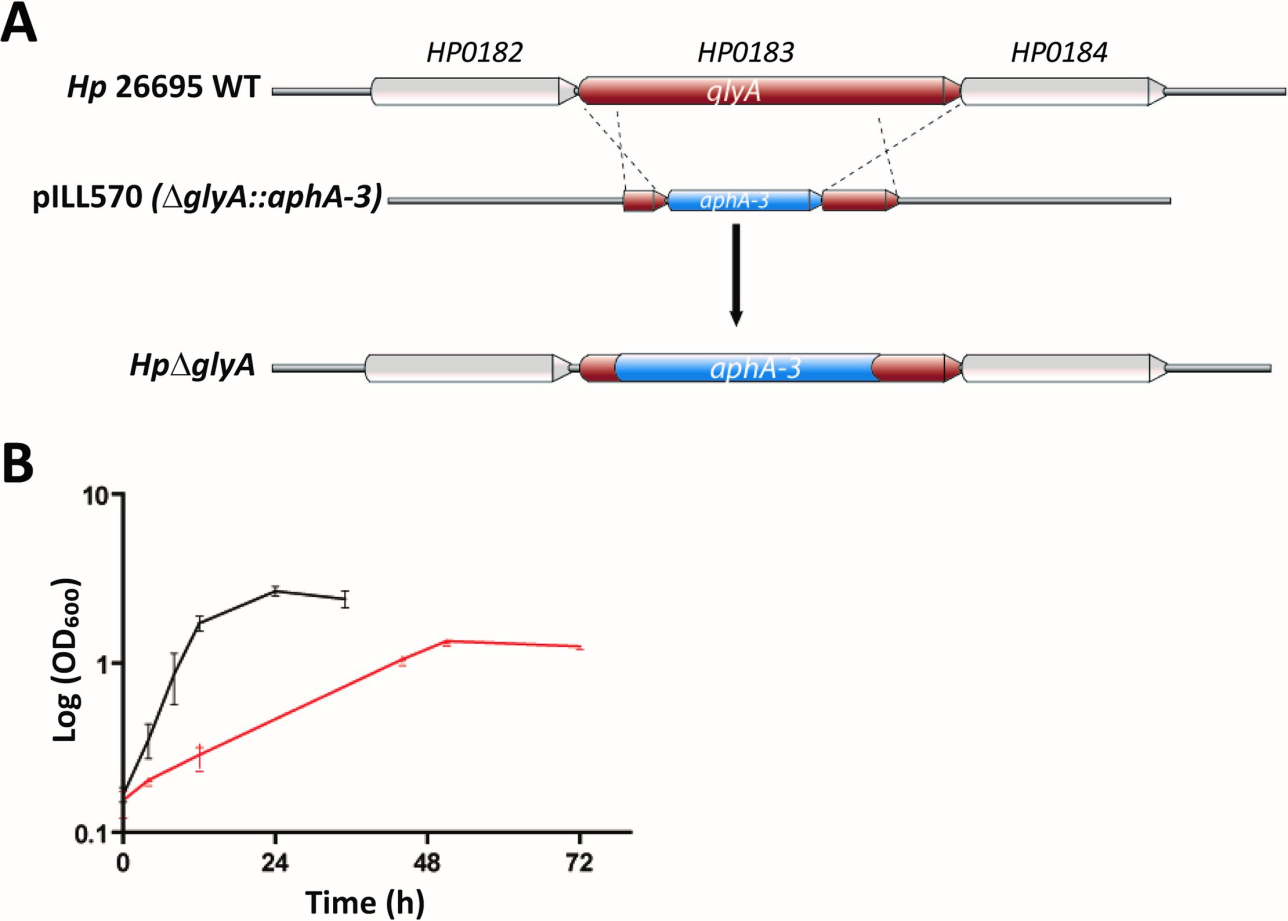
**(A)** Schematic representation of *glyA* (HP0183) inactivation in *H*. *pylori*. *glyA*, coding for SHMT, was partially deleted and replaced with a kanamycin resistance cassette resulting in plasmid pILL570 (*glyA*::*aphA-3*). *H*. *pylori* 26695: WT strain; *Hp*Δ*glyA*: *glyA* deletion strain. **(B)** Growth curves of strains *H*. *pylori* 26695 WT (black curve) and *Hp*Δ*glyA* (red curve) in BHI medium supplemented with 10% fetal calf serum. Each point represents the mean of three independent measurements.

To improve our understanding of the physiological role of *H*. *pylori* SHMT, we inactivated the *glyA* gene in the parental strain *Hp*26695, resulting in the deletion strain *Hp****Δ****glyA*. For inactivation of *glyA*, the strain *Hp*26695 was transformed with plasmid pILL570 (*glyA*::*aphA-3*), and a non-polar kanamycin resistance cassette was inserted into the *glyA* gene of *H*. *pylori* by homologous recombination. A schematic representation of this inactivation is presented in **Fig 6A**.

The *H*. *pylori* ***Δ****glyA* deletion strain shows growth both on solid medium and in liquid cultures, although with a growth rate considerably slower compared to the wild-type strain *Hp*26695 under the same conditions. In liquid cultures we measured a doubling time of 21 hours for *Hp****Δ****glyA*, compared to four hours for the WT strain *Hp*26695 (**Fig 6B).** Altogether, our findings demonstrate that *glyA* codes for crucial metabolic functions in *H*. *pylori*, as exemplified by considerably impaired cellular growth.

**Fig 7:**
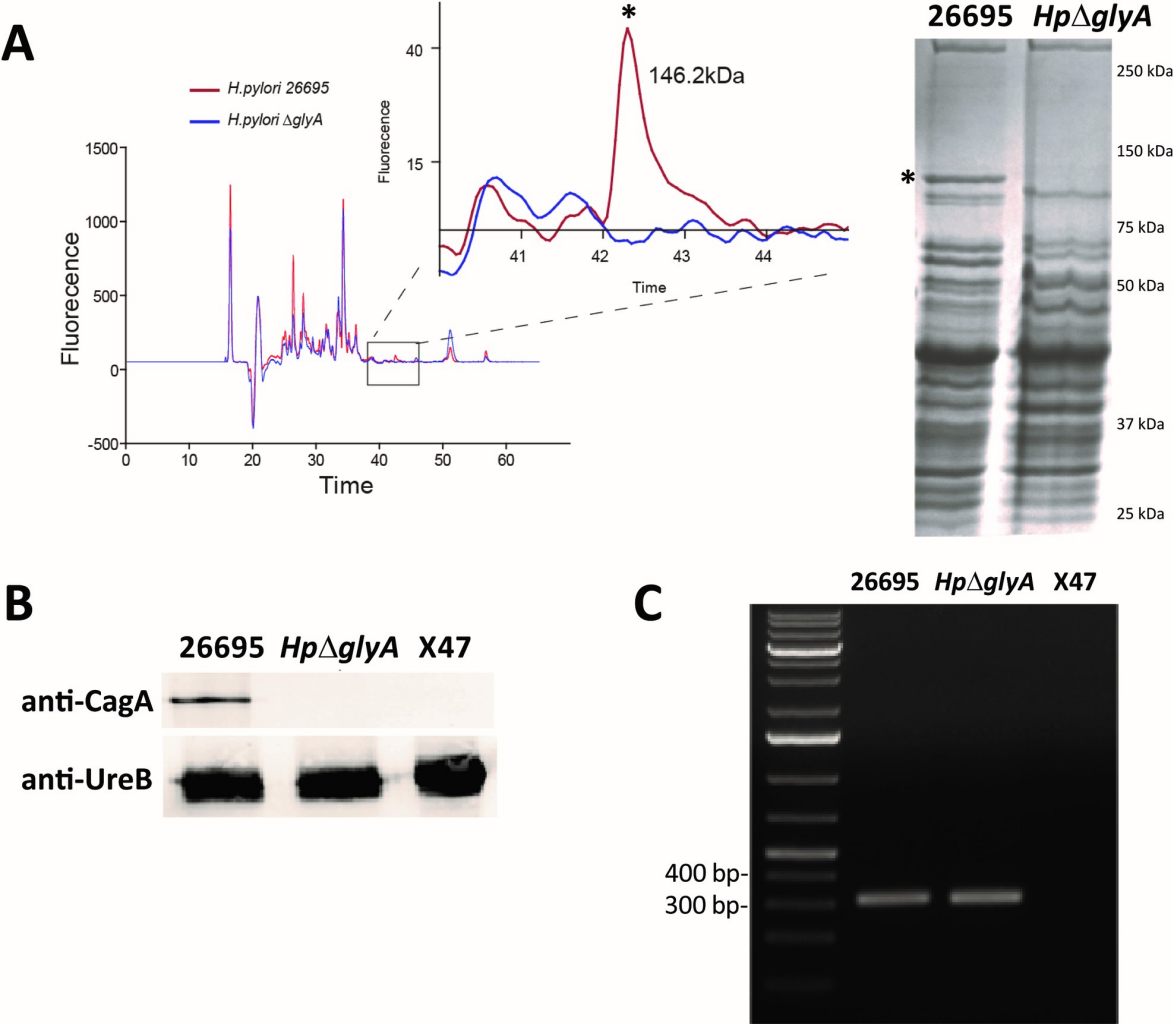
**(A)** Comparison of the overall protein profiles in crude cell extracts of wild-type *H*. *pylori* 26695 (red) and the *Hp*Δ*glyA* deletion strain (blue) as determined by capillary electrophoresis (Experion, Bio-Rad) (left) and by migration on SDS-PAGE (right). The absence of a discrete protein band (*) with a molecular mass around 145 kDa is observed in the *ΔglyA* strain. **(B)** Immunoblot of crude extracts of *H*. *pylori* strains: 26695 (*glyA*^+^, *cagA*^+^), *Hp*Δ*glyA* (*glyA*^-^, *cagA*^+^) and X47 (*glyA*^+^, *cagA*^-^). Immunodetection was performed with antibodies directed against CagA and UreB proteins from *H*. *pylori*. **(C)** PCR amplification of a 307 bp internal fragment of *cagA* performed with the primer pair SA82/SA83 (**Table 1**). Amplification is observed in the wild-type 26695 and *Hp*Δ*glyA* deletion strains, but not in the X47 control strain.

To obtain further insight into the properties of the *Hp****Δ****glyA* deletion strain, we compared the overall protein profiles in crude cell extracts of *H*. *pylori* WT with the Δ*glyA* strain at different growth times. Using two independent experimental methods, we observed the loss of a polypeptide with an apparent molecular mass of about 145 kDa in *Hp****Δ****glyA* (note that the molecular mass of *Hp*SHMT is 45 kDa) (**Fig 7A**). The band corresponding to the unknown protein with altered expression level was subjected to chymotryptic digests and mass spectrometric analyses (MALDI-TOF) and was identified as CagA, the principal virulence factor in *H*. *pylori*, with a molecular mass of 146 kDa. The peptides that were identified by mass spectrometry and attributed to CagA are listed in **S2 Table**. Immuno-detection analysis with monoclonal antibodies directed against the CagA protein confirmed the absence of CagA in crude extracts of the *Hp****Δ****glyA* strain (**Fig 7B**). A 307 bp DNA fragment that corresponded to an internal region of *cagA* was amplified in the WT strain *Hp*26695 (*glyA*^+^*cagA*^+^) and in *Hp****Δ****glyA* (*glyA*^-^ *cagA*^+^). No amplification was observed in the type II control strain X47 (*glyA*^*+*^*cagA*^*-*^) [62, 63] (**Fig 7C**).

Systematic PCR amplifications showed that the promoter region and the 5’ region of *cag*A were not amplified in the *Hp****Δ****glyA* strain (**S2A Fig**). Deep sequencing of the complete genome of *Hp****Δ****glyA* using IonTorrent technology strikingly revealed the full deletion of a 27,716 bp region (from coordinate 552,383 to 580,099) (**S2B Fig**). This deletion extends from the *cag5* gene to the *cagA* gene, with the loss of the 178 first nucleotides of *cagA*. Note that this region was present in the 26695 parental strain used to construct the aforementioned deletion strain, as confirmed by whole genome sequencing. These data explain the absence of PCR amplification with the primer pair SA129/SA83 and consequently the lack of production of the CagA protein in the *Hp****Δ****glyA* strain. Altogether ≈ 77% of the *cag* pathogenicity island (*cag*PAI) are lost in the *Hp****Δ****glyA* deletion strain (**S2 Fig**).

## Discussion

Here, functional *in vivo* complementation of an *E*. *coli* Δ*glyA* strain demonstrated that the *Helicobacter pylori* HP0183 gene product displayed serine hydroxymethyltransferase activity (**Fig 2**). The physiologically relevant cellular reaction of SHMT is the reversible interconversion of serine and THF to glycine and MTHF [1–3]. This reaction is the principal source of cellular glycine, so consequently loss of SHMT activity has been correlated with glycine auxotrophy in *E*. *coli* [44, 45] and humans [46]. Importantly, the SHMT-catalysed reaction results in formation of MTHF, which is one of the few C1 donors in biosynthesis. MTHF is subsequently utilized for thymidylate, methionine or purine biosynthesis. Interestingly, in many organisms including animals, plants and bacteria, a glycine-inducible glycine cleavage system is present that provides an alternative biosynthetic route for the generation of MTHF. It is part of the most prominent glycine and serine catabolism pathway and, when coupled to SHMT, catalyzes the following reversible reaction: 2 glycine + NAD^+^ + H_2_O → serine + CO_2_ + NH_3_ + NADH + H^+^. Here the methyl group derived from the catabolism of glycine can be transferred to other key molecules, such as purines and methionine. Based upon the KEGG database [20, 21], in organisms containing ThyX as the only thymidylate synthase, as is the case for *H*. *pylori*, SHMT appears to be the only enzyme capable of synthesizing MTHF from THF.

*Hp*SHMT binds its cofactor PLP only weakly, essentially resulting in the loss of PLP during protein purification, but is able to form a stable ternary complex with PLP in the presence of substrates/products, as demonstrated biochemically and spectroscopically (**Fig 3B and 3C**). No PLP was present in the *Hp*SHMT structure (**Fig 4**), providing one of the very few apoprotein structures of an SHMT enzyme. Superposition of the *Hp*SHMT apoprotein with *Tt*SHMT and *Ec*SHMT reveals marked differences at the active site that is almost completely disordered in *Hp*SHMT. As a more detailed view of the active site (**Fig 5A**) indicates a very strong structural conservation of the residues involved, we propose that the structure of *Hp*SHMT represents an inactive conformation of the enzyme prior to PLP binding. Two consecutive glycine residues that precede the PLP-stacking histidine residue are conserved across bacterial SHMT sequences and are located in a glycine rich loop. In the *Hp*SHMT apoprotein these residues (G121, G122) are located in the disordered loop 2 (residues 117–137). This was found to be the case also for the equivalent glycine-rich loops of apo-SHMT of the psychrophilic bacterium *Psychromonas ingrahamii* [53] and in the X-ray structure of apo-SHMT from *Salmonella typhimurium* [54]. It has been suggested that the flexibility of this loop may be essential for the PLP cofactor uptake mechanism, which may be possibly shared by most bacterial SHMTs [53]. In the apo-SHMTs, the loop containing the invariant histidine residue (His123 in *Hp*SHMT) that makes a stacking interaction with the pyridine cofactor ring is disordered, indicating the pivotal role of this interaction in the structural rearrangement occurring upon cofactor binding (**Fig 5A**). These findings are in agreement with earlier studies indicating that the PLP cofactor binds to the already folded dimeric apo-SHMT [64]. In bacterial type I PLP-dependent enzymes [65, 66] and the human enzyme DOPA decarboxylase [67], binding of PLP was found to induce folding and rearrangement of loops located around the active site. Thus the folding of specific loops is a prerequisite for PLP binding and in agreement with our proposal that the *Hp*SHMT structure represents an inactive conformation. This is analogous to what has been previously proposed for thymidylate synthase ThyA that has been shown to exist in active and inactive loop configurations [68]. Note that the stabilization of the proposed inactive configuration using small molecules may provide a specific way for inhibiting *Hp*SHMT.

The *H*. *pylori* ***Δ****glyA* deletion strain shows growth on both solid media and in liquid cultures, although with a growth rate considerably slower compared to the parental wild-type strain *Hp*26695 (**Fig 6B**). At first sight, the non-essential nature of the ubiquitous SHMT enzyme might appear surprising. However, a genomic study mapping the location of transposon (Tn) insertions in *H*. *pylori* showed one Tn hit in *glyA* [69], supporting the idea that *glyA* is not essential. In Leishmania, in serine rich medium, the *glyA* genes are dispensable for growth, but when the cells are grown in poorer medium, the *glyA* deletion mutants appear auxotroph for serine [70]. In *E*. *coli*, multicopy suppression of Δ*glyA* by the isoenzyme LtaE (L-*allo*-threonine aldolase) has been described [71, 72]. Moreover, rescue of *glyA* mutants was observed in a pathway catalysed by the *tdh* and *kbl* gene products, threonine dehydrogenase and glycine C-acetyltransferase, respectively [72, 73]. Analysis of the KEGG database [20, 21] revealed the presence of the respective homologs of these proteins in *H*. *pylori* 26695, inviting further investigation. Altogether, this suggests that these potential alternative pathways likely exist in *H*. *pylori*, but that they are considerably less efficient than *Hp*SHMT itself.

Our findings highlight the crucial metabolic functions of SHMT in *H*. *pylori*, as exemplified by the considerably impaired cellular growth of the *glyA* deletion strain. Together with thymidylate synthase ThyX, SHMT is crucial for *de novo* synthesis of thymidylate (dTMP) in *H*. *pylori* (**Fig 1**). Inadequate *de novo* thymidylate biosynthesis is known to slow down DNA replication and increase genome instability. For example, *de novo* thymidylate biosynthesis activity was found to be reduced by 75% in nuclei isolated from *shmt1* knockout mice [74]. Mice lacking SHMT1 are vital, but have an abnormal accumulation of uracil in DNA [75]. In lung cancer cells, knockdown of SHMT1 induces apoptosis as a result of uracil misincorporation during DNA replication and a decrease in dTMP synthesis [76]. It it thus reasonable to propose that metabolically challenged *H*. *pylori* Δ*glyA* cells may replicate their chromosomal DNA even slower than wild-type cells [19], resulting in replication stress known to be linked to chromosomal instability [77, 78].

Protein profiling together with PCR and deep sequencing approaches showed the loss of ≈ 77% of *cag*PAI [79] in the characterized *Hp*Δ*glyA* deletion strain (**Fig 7**). Although this observation *per se* does not imply causation and *H*. *pylori* strains are known to exhibit a high level of genetic diversity [80, 81], it is of interest to note that the *glyA* deletion strain shows no chromosomal changes in the known genome plasticity regions I and II [82]. Further analyses of independent deletion mutants will be required to investigate a potential direct link between the deletion of *glyA* and the loss of *cag*PAI.

## Conclusions

In the present study we identified and characterized the enzyme SHMT from the human pathogenic bacterium *H*. *pylori*. Its activity was confirmed by functional complementation assays, supported by biochemical studies. A *H*. *pylori ΔglyA* strain was viable, but exhibited markedly slowed growth compared to wild type and lacked CagA. The possibility of a direct link between the *glyA* deletion and genomic stability in *H*. *pylori* clearly mandates further studies. The three-dimensional structure of the *H*. *pylori* SHMT apoprotein provided insight into the low affinity of the enzyme for its PLP cofactor and revealed marked differences in loop configurations at the active site. It is of note that the stabilization of the proposed inactive configuration using small molecules may provide a specific way for inhibiting *Hp*SHMT.

## Supporting information

S1 Fig Partial deletion and chromosomal replacement of *glyA* in *H. pylori* 26695. (A) Partial deletion and chromosomal replacement of the *glyA* gene by a non-polar cassette *aphA-3* (Kn^R^) in *H. pylori* 26695. The chromosomal organization of the 26695 wild type and the resulting *ΔglyA* mutant strain is shown together with the adjacent genes HP0182 and HP0184. The small black arrows indicate primers used in this study for verification of the correct allelic replacement of *glyA* with *aphA-3*. (B) PCR amplification reactions to verify the allelic replacement of *glyA* with *aphA-3*. Expected sizes: SK40/oEF23: T, 0.70 kbp and *ΔglyA*, no amplification; oEF22/SK41: T, 1.46 kbp and *ΔglyA*, 1.65 kbp; oEF22/oEF23: T, 0.30 kbp and *ΔglyA*, no amplification; H17/SK41: T, no amplification and *ΔglyA*, 0.71 kbp. (C) Artemis genome browser screenshot confirming the deletion of *glyA* in the *ΔglyA* mutant strain.
(PDF)

S2 Fig Loss of 77% of *cagPAI* in *H. pylori ΔglyA*. **(A)** PCR amplification of *cagA* internal regions with different sets of primers. Oligonucleotides are represented by blue arrows. The red box corresponds to the 178 bp sequence deleted at the 5’ extremity of the *cagA* coding sequence in the *Hp****Δ****glyA* mutant. The numbers, flanking the black arrow representing the *cagA* gene and the red box, correspond to the genomic coordinates in *H*. *pylori* 26695 (NC_000915.1). Wt: wild type and mt: ***Δ****glyA* mutant. **(B)** Artemis genome browser screenshots illustrating the coverage overview of the 26995 wild type and ***Δ****glyA* mutant genomes sequenced on an Ion Torrent PGM. The inset shows the 27,716 bp deletion into the *cag*-PAI in the ***Δ****glyA* mutant (coordinates: 552,383–580,099). The extremities of the gap are located at 80 bp from the 5’ extremity of the *cag5* gene (HP0524) (left end) and at 178 bp from the 5’ extremity of the *cagA* gene (HP0547) (right end).
(PDF)

S1 Table Coverage analysis reports using the *H. pylori* 26695 genome (NC_000915.1) as reference. (PDF)

S2 Table List of peptides identified by mass spectrometry and attributed to the virulence factor CagA. (PDF)
