# Supplementary material for: Structural and functional insight into serine hydroxymethyltransferase from Helicobacter pylori
Source: PLoS One. 2018 Dec 14;13(12):e0208850. doi: 10.1371/journal.pone.0208850 (PMC6294363; doi:10.1371/journal.pone.0208850)
Supplement: S1 Fig — (A) Partial deletion and chromosomal replacement of the glyA gene by a non-polar cassette aphA-3 (KnR) in H. pylori 26695. The chromosomal organization of the 26695 wild type and the resulting ΔglyA mutant strain is shown together with the adjacent genes HP0182 and HP0184. The small black arrows indicate primers used in this study for verification of the correct allelic replacement of glyA with aphA-3. (B) PCR amplification reactions to verify the allelic replacement of glyA with aphA-3. Expected sizes: SK40/oEF23: T, 0.70 kbp and ΔglyA, no amplification; oEF22/SK41: T, 1.46 kbp and ΔglyA, 1.65 kbp; oEF22/oEF23: T, 0.30 kbp and ΔglyA, no amplification; H17/SK41: T, no amplification and ΔglyA, 0.71 kbp. (C) Artemis genome browser screenshot confirming the deletion of glyA in the ΔglyA mutant strain. (PDF) [file pone.0208850.s001.pdf]

S1 Fig

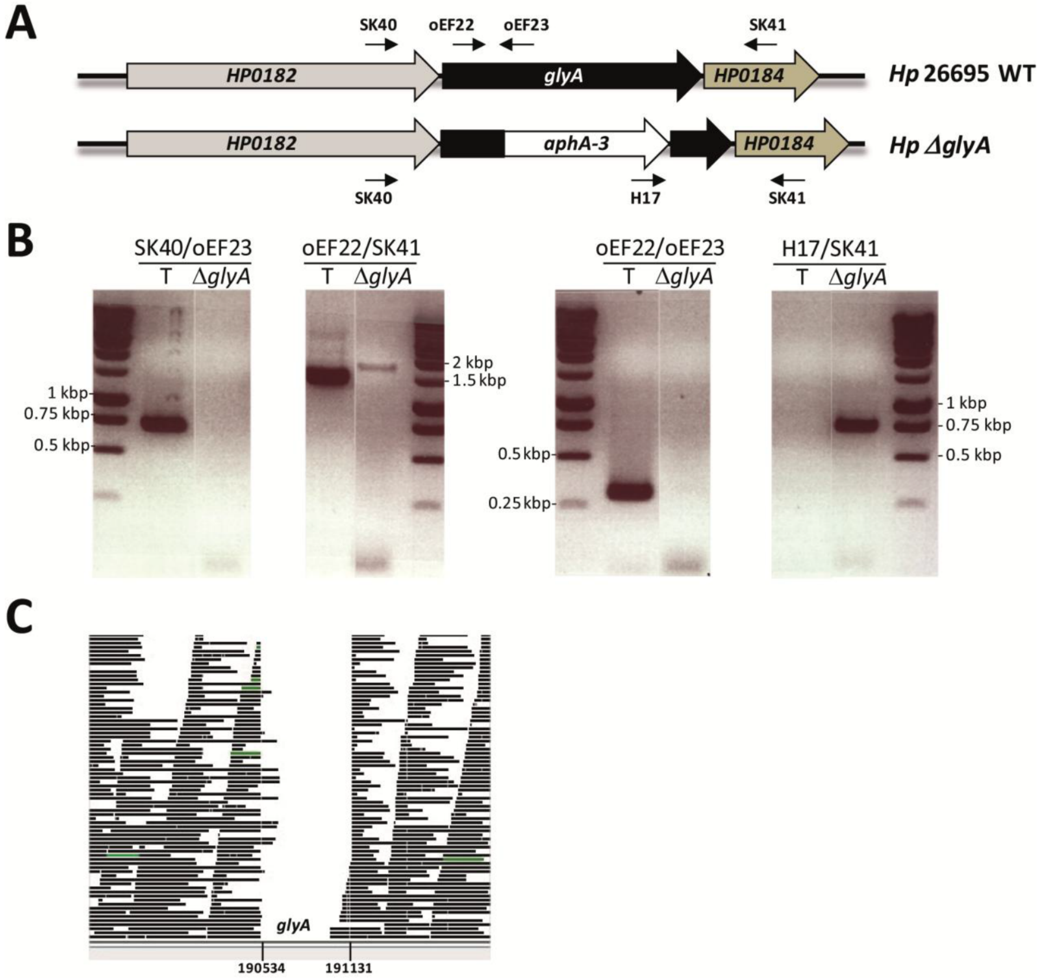

S1 Fig. Partial deletion and chromosomal replacement of *glyA* in *H. pylori* 26695. (A) Partial deletion and chromosomal replacement of the *glyA* gene by a non-polar cassette *aphA-3* (Kn^R) in *H. pylori* 26695. The chromosomal organization of the 26695 wild type and the resulting Δ*glyA* mutant strain is shown together with the adjacent genes HP0182 and HP0184. The small black arrows indicate primers used in this study for verification of the correct allelic replacement of *glyA* with *aphA-3*. **(B)** PCR amplification reactions to verify the allelic replacement of *glyA* with *aphA-3*. Expected sizes: SK40/oEF23: T, 0.70 kbp and Δ*glyA*, no amplification; oEF22/SK41: T, 1.46 kbp and Δ*glyA*, 1.65 kbp; oEF22/oEF23: T, 0.30 kbp and Δ*glyA*, no amplification; H17/SK41: T, no amplification and Δ*glyA*, 0.71 kbp. **(C)** Artemis genome browser screenshot confirming the deletion of *glyA* in the Δ*glyA* mutant strain.
