# Supplementary material for: Structural and functional insight into serine hydroxymethyltransferase from Helicobacter pylori
Source: PLoS One. 2018 Dec 14;13(12):e0208850. doi: 10.1371/journal.pone.0208850 (PMC6294363; doi:10.1371/journal.pone.0208850)
Supplement: S2 Fig — (A) PCR amplification of cagA internal regions with different sets of primers. Oligonucleotides are represented by blue arrows. The red box corresponds to the 178 bp sequence deleted at the 5’ extremity of the cagA coding sequence in the HpΔglyA mutant. The numbers, flanking the black arrow representing the cagA gene and the red box, correspond to the genomic coordinates in H. pylori 26695 (NC_000915.1). Wt: wild type and mt: ΔglyA mutant. (B) Artemis genome browser screenshots illustrating the coverage overview of the 26995 wild type and ΔglyA mutant genomes sequenced on an Ion Torrent PGM. The inset shows the 27,716 bp deletion into the cag-PAI in the ΔglyA mutant (coordinates: 552,383–580,099). The extremities of the gap are located at 80 bp from the 5’ extremity of the cag5 gene (HP0524) (left end) and at 178 bp from the 5’ extremity of the cagA gene (HP0547) (right end). (PDF) [file pone.0208850.s002.pdf]

S2 Fig

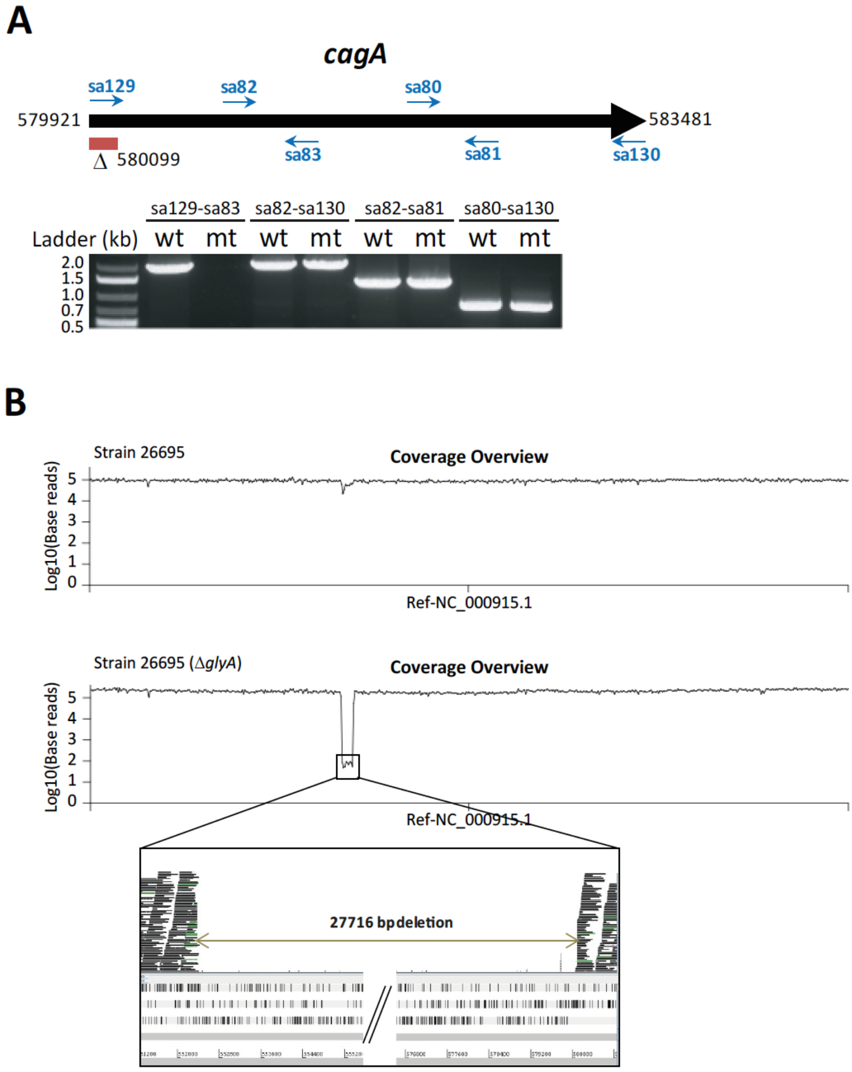

S2 Fig. Loss of 77% of *cagPAI* in *H. pylori* Δ*glyA*. (A) PCR amplification of *cagA* internal regions with different sets of primers. Oligonucleotides are represented by blue arrows. The red box corresponds to the 178 bp sequence deleted at the 5' extremity of the *cagA* coding sequence in the *Hp*Δ*glyA* mutant. The numbers, flanking the black arrow representing the *cagA* gene and the red box, correspond to the genomic coordinates in *H. pylori* 26695 (NC_000915.1). Wt: wild type and mt: Δ*glyA* mutant. **(B)** Artemis genome browser screenshots illustrating the coverage overview of the 26995 wild type and Δ*glyA* mutant genomes sequenced on an Ion Torrent PGM. The inset shows the 27,716 bp deletion into the *cag*-PAI in the Δ*glyA* mutant (coordinates: 552,383 - 580,099). The extremities of the gap are located at 80 bp from the 5' extremity of the *cag5* gene (HP0524) (left end) and at 178 bp from the 5' extremity of the *cagA* gene (HP0547) (right end).
