# Supplementary material for: Structural and functional insight into serine hydroxymethyltransferase from Helicobacter pylori
Source: PLoS One. 2018 Dec 14;13(12):e0208850. doi: 10.1371/journal.pone.0208850 (PMC6294363; doi:10.1371/journal.pone.0208850)
Supplement: S1 Table — (PDF) [file pone.0208850.s003.pdf]

S1 Table. Coverage analysis reports using the *H. pylori* 26695 genome (NC_000915.1) as reference.

Characteristics	Sample	
	**Wild type**	***H. pylori*_Δ*glyA***
**Number of mapped reads**	219317	476251
**Percent reads on target**	100.00%	100.00%
**Number of contigs**	1	1
**Average base coverage depth**	32.06	76.78
**Total base reads on target**	53469632	128064498
**Uniformity of base coverage**	99.72%	98.05%
