# Supplementary material for: Structural and functional insight into serine hydroxymethyltransferase from Helicobacter pylori
Source: PLoS One. 2018 Dec 14;13(12):e0208850. doi: 10.1371/journal.pone.0208850 (PMC6294363; doi:10.1371/journal.pone.0208850)
Supplement: S2 Table — (PDF) [file pone.0208850.s004.pdf]

S2 Table. List of peptides identified by mass spectrometry and attributed to the virulence factor CagA.

Start	End	Peptide Sequence	Start	End	Peptide Sequence
37	57	(K)VDNVVASFDPDQKPIVDKNDR(D)	500	12	(K)HDGVMFVDYSNFK(Y)
61	70	(R)QAFDGISQLR(E)	523	539	(K)GVGATNGVSHLEAGFNK(V)
61	76	(R)QAFDGISQLREEYSNK(A)	540	558	(K)VAVFNLPDLNNLAITSFVR(R)
85	94	(K)NQYFSDFIDK(S)	651	666	(K)AQANSQKDEIFALINK(E)
95	112	(K)SNDLINKDNLIDVESSTK(S)	674	682	(R)AIAYTQNLK(G)
145	161	(R)NFMENIIQPPIPDDKEK(A)	705	710	(K)SFDEFK(N)
170	182	(K)QSFAGIIIGNQIR(T)	744	755	(K)VENLNAALNEFK(N)
187	196	(K)FMGVFDESLK(E)	770	784	(K)SDLENSVKDVIINQK(V)
231	260	(K)EAINQEPVPHVQPDIATTTTDIQGLPPEAR(D)	838	846	(K)NSELYQSVK(N)
261	266	(R)DLLDER(G)	881	892	(K)FKNFNNNNNGLK(N)
344	359	(K)DQQGNNVATLINVHMK(N)	907	923	(K)TGQVASPEEPIYTQVAK(K)
372	382	(K)GINNPSFYLYK(E)	929	951	(K)IDRLNQIASGLGGVGQAAGFPLK(R)
372	391	(K)GINNPSFYLYKEDQLTGSQR(A)	932	952	(R)LNQIASGLGGVGQAAGFPLKR(H)
383	391	(K)EDQLTGSQR(A)	962	985	(K)VGLSASPEPIYATIDDLGGPFPLK(R)
392	399	(R)ALSQEEIR(N)	962	986	(K)VGLSASPEPIYATIDDLGGPFPLKR(H)
424	435	(K)EKFQNEIEDFQK(D)	1020	1032	(K)AGFFGNLEQTIDK(L)
426	435	(K)FQNEIEDFQK(D)	1020	1034	(K)AGFFGNLEQTIDKLK(D)
426	438	(K)FQNEIEDFQKDSK(A)	1060	1070	(K)LDNYAINSHTR(I)
439	448	(K)AYLDALGNDR(I)	1103	1118	(K)IVAHNVGSVSLSEYDK(I)
459	476	(K)HSALITEFNNGDLSYTLK(D)			
